# Binge-eating disorder diagnosis and treatment: a recap in front of DSM-5

**DOI:** 10.1186/s12888-015-0445-6

**Published:** 2015-04-03

**Authors:** Federico Amianto, Luisa Ottone, Giovanni Abbate Daga, Secondo Fassino

**Affiliations:** Department of Neurosciences, University of Torino, Psychiatric Clinic, Via Cherasco 11, 10126 Turin, Italy

**Keywords:** Binge eating disorder, Overeating, Obesity, Eating disorders, Treatment

## Abstract

**Introduction:**

Binge Eating Disorders is a clinical syndrome recently coded as an autonomous diagnosis in DSM-5. Individuals affected by Binge Eating Disorder (BED) show significantly lower quality of life and perceived health and higher psychological distress compared to the non-BED obese population. BED treatment is complex due to clinical and psychological reasons but also to high drop-out and poor stability of achieved goals. The purpose of this review is to explore the available data on this topic, outlining the state-of-the-art on both diagnostic issues and most effective treatment strategies.

**Methods:**

We identified studies published in the last 6 years searching the MeSH Term “binge eating disorder”, with specific regard to classification, diagnosis and treatment, in major computerized literature databases including: Medline, PubMed, PsychINFO and Science Direct.

**Results:**

A total of 233 studies were found and, among them, 71 were selected and included in the review.

**Review:**

Although Binge Eating Disorder diagnostic criteria showed empirical consistency, core psychopathology traits should be taken into account to address treatment strategies. The available body of evidence shows psychological treatments as first line interventions, even if their efficacy on weight loss needs further exploration. Behavioral and self-help interventions evidenced some efficacy in patients with lower psychopathological features. Pharmacological treatment plays an important role, but data are still limited by small samples and short follow-up times. The role of bariatric surgery, a recommended treatment for obesity that is often required also by patients with Binge Eating Disorder, deserves more specific studies. Combining different interventions at the same time does not add significant advantages, planning sequential treatments, with more specific interventions for non-responders, seems to be a more promising strategy.

**Conclusions:**

Despite its recent inclusion in DSM-5 as an autonomous disease, BED diagnosis and treatment strategies deserve further deepening. A multidisciplinary and stepped-care treatment appears as a promising management strategy. Longer and more structured follow-up studies are required, in order to enlighten long term outcomes and to overcome the high dropout rates affecting current studies.

## Introduction

The fifth edition of Diagnostic and Statistical Manual of Mental Disorders (DSM-5) [1] published in May 2013, codes for the first time Binge Eating Disorder (BED) as an autonomous Eating Disorder (ED), while before it was listed as an Eating Disorder not Otherwise Specified (EDNOS) needing future definition. This disease is of particular interest for its frequency in primary care, its link with obesity and its medical and psychiatric comorbidities, leading to high socio-economic impact due to reduced quality of life and increased health service utilization [2,3]. It is in fact a relatively common disorder, with an estimated lifetime prevalence in general population around 1.4% [4]. However, this estimate substantially increases among obese individuals with no marked gender differences [4]. Prevalence is likely to increase, especially because of the rising incidence that both obesity and EDs show worldwide rather than for the widening of diagnostic criteria introduced in DSM-5 [5,6].

Though BED was initially considered a disorder of adulthood, recent studies furthermore suggest a lower age of onset than that previously assumed [4]. Increasing evidence is emerging that it already occurs in adolescence and childhood, with an average age of onset ranging from late teens to early 20s and a mean persistence of 4.3 years [4]. An earlier onset of bingeing seems to predict worse outcome and to require more complex interventions [7]. In addition, although there is still a wide debate on the clinical stability of such a diagnosis, data showed that it tends to be a stable syndrome, with relative stability of binge eating patterns [8] and lower crossover rates than other EDs [9].

The clinical importance of BED is also related to its frequent comorbidity with obesity as well as complications of overweight [4] and with psychiatric symptoms like depression and anxiety, frequently linked to excessive concerns about food, body shape and weight [10,11]. Moreover, like other EDs, BED patients show relevant alexithymia and deficit in emotional identification and regulation [12,13] and high interpersonal problems [14,15]. Personality disorders are also frequently diagnosed among BED patients and mood disorders and substance abuse are frequent comorbidities [4,16], eventually related to more severe psychopathology and worse prognosis [17-19]. These correlations are so relevant that these comorbidities have been proposed as markers of major severity, rather than just associated conditions [19].

Also due to these relevant comorbidities, perceived quality of life and health are significantly lower in BED than in the non-BED obese population, with great expression of depressive and negative feelings, disinhibition and anger [11,13,20]. Low mood and negative feelings seem to be related to severe eating impulsiveness, leading to significant psychopathological impairment even in presence of a limited food assumption [13,18]. Eating impulsiveness itself has been related to greater physical discomfort and worse quality of life, independently from Body Mass Index (BMI) and objective functional impairment, and it is also consequently related to higher treatment seeking [15,20,21].

The clinical relevance of BED, here underlined, highlights the importance of identifying appropriate and evidence based treatment strategies, in order to prevent this disorder to become chronic and complicated [4]. It is the purpose of this review to explore the state-of-the-art on this topic, exposing current evidence on diagnosis and treatment.

## Methods

We aimed to systematically identify and synthesize the recent evidences about BED diagnostic category with regards to its recent addition as an autonomous disease in DSM-5, with a focus on its clinical features, psychopathological correlates and treatment evidences. In relation to these issues, we summarized the principal findings published from January 2008 to May 2014 about this topic. We choose this time frame because the first articles claiming BED inclusion in DSM-5 in online databases date back this year [22]. We identified relevant studies searching the MeSH Term “binge eating disorder”, with specific regard to classification, diagnosis and treatment, in major computerized literature databases including: Medline, PubMed, PsychINFO and Science Direct. We focused on clinical studies about adult population, excluding studies about children and young adults. We also excluded from the evaluation case reports and case series studies.

Any experimental research that is reported in the manuscript has been performed with the approval of an appropriate ethics committee. Research carried out on humans are in compliance with the Helsinki Declaration, and experimental research on animals follows internationally recognized guidelines.

## Results

A total of 233 studies were found and 71 were included in this review according to the aforementioned criteria (see Table 1 for a summary). To enhance the specificity of our search, we considered only studies based on the specific diagnosis of BED. We therefore excluded 64 studies pertaining to mixed ED samples, non-purging bulimia nervosa, atypical EDs, EDNOS, if not including BED, physical comorbidities of binge eating and obesity or the ICD-10 category “overeating associated with other psychiatric condition”. We also excluded 24 studies referring to sub-threshold BED, a clinical category meant to be redefined by the application of diagnostic criteria for EDs in DSM-5.

**Table 1:** Summary of studies included in the review*

Thematic area	Article n.	Authors	Patients n./ *Articles n.*	Duration (months)	Drop-out rates	Main results	Type of study
Diagnostic issues and pathological features	23	Bautista-Diaz et al., 2012 [21]	70 obese women (35 BED; 35 non BED)	-----	-----	Higher body dissatisfaction and stronger influence of socio-cultural factors on eating psychopathology (at self-administered test) in women with BED vs controls.	Case–control study
		Blomquist et al., 2011 [49]	78 obese with BED	-----	-----	Mean weight gain of 15.1 pounds during the year before treatment in BED patients (structured interviews + self-administered test). Weight change associated with more frequent binge eating and overeating during breakfasts.	Retrospective observational study
		Blomquist et al., 2012 [14]	84 obese with BED	-----	-----	Higher interpersonal problems (at interpersonal circumplex structural summary method) observed in in BED patients and associated with earlier onset of binges and overweight.	Cross-sectional study
		Carano et al., 2012 [12]	80 BED	-----	-----	Among BED patients, 27.5% refer suicide ideation and 12.5% previous attempts of suicide (self-administered tests). Alexithymia related to higher suicide ideation and previous suicide attempts.	Cross-sectional study
		Carrard et al., 2012 [18]	92 women with BED (full-criteria and subthreshold)	6 treatment	10.6 – 30.8%	Participants to an internet self-help program split by cluster analyses into pure dietary subtype (71.7%) and dietary-negative affect subtype (28.3%) (clinical interview + self-administered tests). Dietary-negative affect subtype show higher frequency of binges, more severe eating disorders, higher tendency to act rashly in the context of negative affect, greater sensitivity to punishment and higher dropout rates (30.8% vs 10.6%).	Case–control study
		Compare et al., 2012 [13]	150 obese BED; 150 obese non BED; 150 healthy controls	-----	-----	Lower mindfulness scores (Five Facet Mindfulness Questionnaire) in BED patients than in healthy and obese controls. Mindfulness negatively correlated with binge frequency, body uneasiness and eating impulsivity (self-administered tests).	Case–control study
		Folope et al., 2012 [20]	130 obese	-----	-----	Presence of EDs impaired significantly quality of life and is related to higher anxiety and depression rates (self-administered tests).	Cross-sectional study
		Gianini et al., 2013 [30]	326 obese with BED	-----	-----	Difficulties with emotion regulation accounted for unique variance in both emotional overeating and general eating pathology (self-administered tests).	Cross-sectional study
		Grilo et al., 2009 [27]	436 (195 BED with overvaluation; 129 BED subclinical overvaluation; 61 BN; 51 sub-threshold BN)	-----	-----	Clinical overvaluation of body shape and weight present in 60% of BED and related to significantly higher levels of eating disorder psychopathology. BED clinical overvaluation group show higher eating concern, shape concern, and weight concern than other groups. This feature warrants consideration either as a diagnostic specifier or as a dimensional severity rating.	Case–control study
		Grilo et al., 2013^1^ [10]	90 BED (52 with overvaluation; 38 without overvaluation)	6 treatment + 12 follow-up	24% (CBT) - 31% (BWL)	Participants randomly assigned to group CBT or BWL. Patients with overvaluation of weight and body shape had significantly greater levels of ED psychopathology, higher depression and lower self-esteem (semi-structured interviews + clinical measures). Overvaluation of shape/weight predicts non-remission from BED and higher frequency of binge eating at 12-month follow-up.	Prospective study
		Hudson et al., 2012 [5]	888 first-degree relatives from a family study of BED	-----	-----	Using the proposed DSM-5 diagnostic criteria vs DSM-IV criteria (clinical interview) will likely have only a minimal effect on global BED prevalence.	Prevalence study
		Masheb et al., 2011a [32]	311 women (39 BN; 69 BED; 203 controls)	-----	-----	Atypical eating patterns (e.g. nibbling, eating double meals and nocturnal eating) more frequent among EDs (at internet questionnaire). Breakfast consumption associated with lower BMI in BED and controls and more frequent meal consumption associated with less binge eating in BED.	Case–control study
		Masheb et al., 2013 [50]	130 obese with BED	-----	-----	83% of treatment seeking obese BED patients gained weight in year before treatment (structured interview + self-report questionnaires). No differences among weight gainers and weight maintainers/losers in current weight and eating behaviors.	Retrospective observational study
		Munsch et al., 2012a [31]	22 women with BED	-----	-----	During binge days (at 1 week of ecological assessment) negative mood and tension are higher and significantly increased at first binge episode, while positive mood strongly and significantly decreased. No indications of accumulation of negative mood triggering binges followed by reinforcing mechanisms in terms of improvement of mood, unlikely to BN.	Cross-sectional study
		Ojserkis et al., 2012 [28]	116 BED (84 with overvaluation, 31 without overvaluation; 1 excluded)	4 treatment	52.25%	Patients selected from a 16-week randomized controlled trial of BWL + individual CBT and/or fluoxetine**. Patients with overvaluation of weight and body shape show higher pre-treatment scores on depression and eating psychopathology and lower self-esteem (self-administered tests). At treatment termination patients with overvaluation still display high binge eating severity.	Prospective observational study
		Peterson et al., 2012 [8]	188 EDs (71 AN, 95 BN, 122 BED)	2 follow-up	0%	Although longitudinal patterns of binge types are variable among individuals with EDs, consistency in objective and subjective binge episodes is most commonly observed.	Retrospective observational study
		Sawaoka et al., 2012 [15]	113 overweight or obese with BED	-----	-----	Social anxiety positively and significantly related with shape and weight concerns and binge frequency (semi structural clinical interviews + self-report measure). Social anxiety and self-consciousness account for significant variance in eating, shape and weight concerns and ED severity but are not associated with BMI or dietary restraint.	Cross-sectional study
		Schag et al., 2013 [16]	51 articles	-----	-----	*Evidences of increased food-related impulsivity, coupled with increased reward sensitivity, in BED patients. BED could represent a specific phenotype of obesity with increased food-related impulsivity.*	*Literature review*
		Striegel-Moore & Franko, 2008 [22]	-----	-----	-----	*Clinical utility and validity of BED diagnostic criteria is consistent and allow BED inclusion in DSM-V.*	*Literature review*
		Thomas et al., 2009 [25]	125 articles	-----	-----	*EDNOS did not differ significantly from AN and BED on eating or general psychopathology while BN show greater eating and general psychopathology. Subthreshold BN or BED did not differ significantly from full syndrome cases.*	*Meta-analysis*
		Trace et al., 2012 [6]	13295 female twins	-----	-----	Lifetime prevalence of BN and BED increased linearly as the frequency criterion for binges decreased. As required duration increased, BED prevalence decreased slightly.	Prevalence study
		White & Grilo, 2011 [26]	916 community volunteers (164 BED, 83 BN, 668 healthy controls)	-----	-----	All of the BED diagnostic criteria in DSM.IV have predictive value. Eating alone because embarrassed and feeling disgusted performed as the best inclusion and exclusion criteria, respectively. Eating when not hungry and eating alone because embarrassed are the best overall indicators for binge eating.	Cross sectional study
		Wolfe et al., 2009 [33]	33 articles	-----	-----	*Majority of binge episodes typically occur in less than 2 h, with size variability across BN and BED but a clinical importance likely related to objective food intake and an increased consumption of carbohydrates and fats. Loss of control is a core feature associated with higher depression, higher body dissatisfaction and poorer related quality of life. Negative affect is the most widely reported binge antecedent.*	*Literature review*
Global treatment	7	Brambilla et al., 2009 [35]	30 (+5 dropped-out) BED (3 groups of 10 patients)	6 treatment	14%	Group 1: 1700-kcal diet + CBT + sertraline (50–150 mg/day) + topiramate (25–150 mg/day); group 2: diet + CBT + sertraline; group 3: nutritional counselling + CBT. Clinical interview and self-administered tests. Binge frequency and body weight decrease only in group 1. Group 2 improved in depression, interpersonal relationship and schizoid personality subscales.	Clinical trial
		Deumens et al., 2012 [59]	212 BED	5 treatment	14%	Higher social embedding and higher openness predict better outcomes at CBT (self-administered tests). Higher depression, agoraphobia and extraversion predict poorer outcome.	Prospective observational study
		Flukinger et al., 2011 [37]	78 BED	4 treatment; 6 follow-up	36%	Low self-esteem predicts premature treatment termination. Low self-esteem experiences, low global alliance, low mastery and clarification experiences predict dropout in patients who report discontentment with therapy as reason for premature termination.	Prospective observational study
		Grilo et al., 2011^1^ [52]	125 obese with BED (45 CBT; 45 BWL; 35 CBT + BWL)	4 treatment + 12 follow-up	24 - 40%	At 12-month, binge-eating remission rates of 51% (CBT), 36% (BWL), 40% (CBT + BWL) and mean BMI losses of −0.9%(CBT), −2.1% (BWL) and 1.5% (CBT + BWL). Overall significant percent BMI loss in CBT + BWL attributable to BWL. Binge-eating remission associated with greater percent BMI loss.	Prospective observational study
		Grilo et al., 2012a^1^ [44]	90 obese with BED (45 CBT; 45 BWL)	4 treatment + 12 follow-up	24 - 40%	Rapid response (≥70% binge reduction in 4 weeks) is present in 57% of participants (67% CBT, 47% BWL) and predicts greater improvements across outcomes. CBT patients did comparably well regardless of rapid response in binge eating and eating psychopathology but not in weight loss. BWL patients without rapid response failed to improve further but those with rapid response show greater reductions in binge frequency, eating psychopathology and weight loss.	Prospective observational study
		Vocks et al., 2010 [36]	*38 articles (1973 patients)*	-----	-----	*Psychotherapy and structured self-help, mainly CBT, recommended as first line treatments for BED. Moderately positive effect on binges and depressive symptoms of pharmacotherapy, manly SSRI. Weight-loss treatments demonstrated moderate binge reduction at uncontrolled studies. Combination treatments not show higher effects than single-treatment. Only weight-loss treatment show a considerable weight reduction.*	*Literature meta-analysis*
		Zunker et al., 2010^2^ [45]	179 BED patients (81 rapid responders; 98 non rapid responders)	2.5 treatment	34.6%	Participant randomized to 3 group manual-based CBT (therapist-led, therapist-assisted, self-help). Decrease in binges during treatment predicts clinical outcome. Participants with a 15% reduction in binges at week-one more likely to achieve remission.	Prospective observational study
Behavioral interventions, psychoeducation and psychotherapies	24	Balestieri et al., 2013 [54]	98 patients (54 BED; 44 EDNOS)	2.5 weekly + 8 monthly sessions	6.1% after 10 weeks, − 39.6% at 8 months	Psychoeducational intervention of 10 weekly group sessions. Post-treatment patients who maintained an ED were asked to participate to 2 fortnightly sessions + monthly sessions for 8 months. At 10 weeks 30.6% of patients remitted and all patients showed significant improvements on binge frequency, BMI, bulimic traits, body dissatisfaction, anxiety, depression and alexithymia. At 8 months 41% recovered from ED, with further reduction of binges and improvement of BMI.	Clinical trial
		Carrard et al., 2011 [62]	74 women with BED (37 Group 1; 37 Group 2)	6 treatment + 6 follow-up	19 - 24,3%	Group 1: 6 months online CBT + 6 months follow-up; group 2: 6 months waiting list + 6 months online CBT. Regular e-mail contact with a coach during intervention. After treatment binge eating, drive for thinness, body dissatisfaction and interoceptive awareness significantly improved. Binge episodes, overall eating symptoms and hunger also decreased. Improvements maintained at follow-up. Higher shape concern and higher drive for thinness among dropouts.	Clinical trial
		Castellini et al., 2011 [9]	793 mixed EDs (of which 283 BED)	6 treatment + 72 follow-up	0%	Patients evaluated with clinical interview undergo 6 months individual CBT. At 6-year follow-up, overall recovery rate was 59.2% for BED and 77.2% for subthreshold BED. Crossover rates (DSM-IV) of 8.8% from BED to BN and 10.9% from BN to BED. Relapse rates of 11.4% for BED and 12.1% for subthreshold-BED. Among relapsed patients who changed diagnosis 18.1% of BN developed BED, 18.7% of BED developed BN and 8.7% of subthreshold-BED developed BED.	Prospective observational study
		Compare et al., 2013 [46]	189 obese with BED (63 EFT; 63 CT; 63 DT)	5 treatment + 6 follow-up	27% DT vs. 12.7% EFT vs. 0% CT	Patients randomized to Emotionally Focused Therapy (EFT), Dietary Counseling (DC), and Combined Treatment (CT). DC showed higher dropout rate. Body weight decreased in all three groups. Eating symptomatology decreased with CT and EFT. At follow-up, 71% of CT patients and 46% in EFT had subthreshold eating impulsivity, whereas no participants in the DC group reached this target.	Clinical trial
		Hilbert et al., 2012 [67]	90 BED (45 CBT; 45 IPT)	5 treatment, 48 follow-up	24.7% of treated sample	20 weekly group sessions + 3 individual sessions of CBT or IPT. Long-term BED recovery rates of 52.0% for CBT and 76.7% for IPT. Recovery from subclinical BED 72.0% for CBT and 83.9% for IPT (non-significant difference). BMI stable in both treatments.	Retrospective study
		Iacovino et al., 2012 [43]	*27 articles*	-----	-----	*Individual and group CBT associated with higher binge abstinence rates compared with supportive therapy and BWL. CBT guided self-help have superior outcomes than BWL guided self-help and comparable to IPT in patients with low additional pathology. IPT is the only treatment with comparable long-term outcomes to CBT. Psychodynamic IPT (additional focus on cyclical relational patterns and negative internalizations) comparable to CBT both post-treatment and at 12-month follow-up. DBT shows some promise as BED treatment, but requires further study.*	*Literature review*
		Wilson & Zandberg, 2012 [61]	*24 articles (12 BN; 9 BED, 3 EDNOS)*	*-----*	*-----*	*Literature evidenced CBT guided self-help as a brief, cost-effective treatment for BED, comparable with more complex interventions and not necessarily contra-indicated for patients with comorbid conditions.*	*Literature review*
		Klein et al., 2012 [64]	10 women with BED or BN	4.5 treatment	50%	DBT showed positive preliminary outcomes on binge eating and eating psychopathology.	Descriptive study
		Leombruni et al., 2010 [53]	297 BED patients	2.5 treatment	27%	Group psychoeducational intervention showed efficacy on eating impulsivity and related psychopathology. No significant results on weight and depression. Higher impulsivity correlates with higher drop-out rates.	Clinical trial
		Masheb et al., 2011b [40]	50 obese with BED (25 CBT + ED; 25 CBT + GN)	6 treatment	14%	Patients randomized to CBT + low-Energy-Density diet (CBT + ED) or CBT + General Nutrition counseling not related to weight loss (CBT + GN). 30% of patients achieved at least a 5% weight loss with binge remission rates of 55–75%. No significant differences among treatments. Significantly better dietary outcomes on energy density, and fruit and vegetable consumption at CBT + ED.	Randomized controlled trial
		Masson et al., 2013 [66]	60 BED (30 treatment; 30 wait-list)	3 treatment + 6 follow-up	25%	Self -help based in DBT manual + six 20-min support calls. Post-treatment efficacy on binge remission, quality of life and eating psychopathology at 6-month follow-up (clinical interview + self-report).	Randomized controlled trial
		Munsch et al., 2012a [31]	80 obese with BED (44 CBT; 36 BWL)	4 treatment + 12 extended care + 72 follow-up	Treatment 27.5% (CBT) -25% (BWL); Follow-up 41% (CBT) - 28% (BWL)	Patients randomized to CBT or BWL group intervention. Strong outcome improvement during active treatment but worsening at follow-up, however with residual improvement at 6-year follow-up relative to pretreatment values. Comparable long-term effects between CBT and BWL. Rapid response predicts favorable outcome.	Clinical trial
		Peterson et al., 2009^2^ [63]	259 BED (69 wait-list; 67 self-help; 63 therapist-assisted; 60 therapist-led)	5 treatment; 12 follow-up	26% mean	Therapist-led vs self-help CBT-based intervention. Binge abstinence in 51.7% of therapist-led, 33.3% of therapist-assisted, 17.9% of self-help group and 10.1% of wait-list. No differences in abstinence rates at follow-up. Therapist-led group show more reductions in binge eating at post treatment and follow-up. Treatment completion rates higher in therapist-led (88.3%) and wait-list (81.2%) groups than in therapist-assisted (68.3%) and self-help (59.7%) groups.	Randomized controlled trial
		Ricca et al., 2010 [57]	144 BED and subthreshold-BED (72 individual CBT; 72 group CBT)	5 treatment; 36 follow-up	7%	Individual and group CBT (clinical interview + self-reported tests) show similar long-term response with a significant binge reduction and mild weight reduction. Lower emotional eating and binge eating severity predict full recovery. Overweight during childhood, full blown BED diagnosis and high emotional eating predict treatment resistance.	Prospective randomized controlled trial
		Robinson & Safer, 2012^2^ [7]	101 BED patients (50 DBT; 51 ACGT)	5 treatment	4% DBT 33.3% ACGT	Comparing DBT to active comparison group therapy (ACGT), patients with Avoidant Personality Disorder or earlier onset of overweight and dieting evidenced worsened outcome.	Randomized controlled trial
		Safer et al., 2010^3^ [65]	101 BED patients (50 DBT; 51 ACGT)	5 treatment; 12 follow-up	4% DBT 33.3% ACGT	Group DBT compared to ACGT show significantly lower dropout rate. Post-treatment binge abstinence and reduction achieved more quickly with DBT-BED but no difference at follow-up.	Randomized controlled trial
		Safer & Joyce, 2011^3^ [48]	101 BED patients (50 DBT; 51 ACGT)	5 treatment; 12 follow-up	4% DBT 33.3% ACGT	Rapid responders, especially to group DBT, show higher binge abstinence at end of treatment and 1 year follow-up and also significantly less attrition.	Randomized controlled trial
		Schlup et al., 2010 [58]	76 BED women (40 CBT-L; 36 CBT-S)	2-4 treatment; 12 follow-up	14% (CBT-S) - 35% (CBT-L)	Comparing long and short term CBT - 16 sessions (CBT-L) vs 8 sessions (CBT-S) – both treatments show significant binge reductions. At the end of active treatment, but not at follow-up, better outcomes in CBT-L. Treatment efficacy for rapid responders and individuals with high dietary negative affect differs between CBT-L and CBT-S.	Randomized controlled trial
		Striegel-Moore et al., 2010 [60]	123 patients (59 BED; 13 BN; 51 subthreshold BED/BN) (59 CBT-gsh; 64 TAU)	3 treatment; 12 follow-up	16.3%	CBT based guided self-help (CBT-gsh, 8 sessions) vs treatment as usual (TAU, non-dietary primary care management). At follow-up, CBT-gsh show greater binge abstinence and adjustment but not weight change.	Randomized controlled trial
		Tasca et al., 2012 [41]	95 BED (48 PIP; 47 gCBT)	4 treatment; 6 follow-up	22%	Both group Psychodynamic Interpersonal Psychotherapy (gPIP) and group CBT (gCBT) improve interpersonal problems (self-administered tests). Higher effects of gPIP on patients with Cold/Distant interpersonal problems and attachment avoidance.	Clinical trial
		Vancampfort et al., 2013 [47]	*3 articles (211 BED women)*	*-----*	*-----*	*Aerobic and yoga exercises reduce binges and BMI. Aerobic exercise also reduces depressive symptoms. CBT with aerobic exercise and not CBT alone reduces BMI. CBBT with aerobic exercise is more effective on depressive symptoms than CBT alone.*	*Literature review*
		Vanderlinden et al., 2012 [56]	56 obese with BED	7 treatment; 60 follow-up	----	Weekly group CBT improves eating behaviors, weight and psychopathology up to 3.5 years follow-up.	Prospective randomized controlled trial
		Wilson et al., 2010 [42]	206 BED patients (64 BWL; 66 CBTgsh; 75 IPT)	6 treatment; 24 follow-up	7% IPT; 28% BWL; 30% CBTgsh	Comparing individual BWL, individual IPT and group CBTgsh. Both IPT and CBTgsh resulted in greater binge remission than BWL. Moderators of outcome were self-esteem and eating psychopathology (semi structured interview + self-administered tests). CBTgsh can be a first-line treatment option, with IPT (or full CBT) suitable for patients with low self-esteem and high eating disorder psychopathology.	Clinical trial
		Woolhouse et al., 2012 [39]	43 women (31% BN; 50% BED; 19% subthreshold BN/BED)	2.5 treatment; 3 follow-up	23.25%	Mindfulness + group CBT reduces binge eating, dieting and body image dissatisfaction both at end of treatment and follow-up. Qualitative interviews with 16 patients attributed the efficacy of mindfulness to increasing self-awareness.	Clinical trial
Pharmacological treatments	13	Arbaizar et al., 2008 [74]	*5 articles (528 BED patients)*	*------*	*-----*	*Short-term treatment with topiramate (50-600 mg/day) is more effective than placebo in decreasing binges and weight in both BN and BED. High number of withdrawals and small sample sizes limit the generalizability of this result*	*Literature review*
		Calandra et al., 2012 [71]	30 depressed patients with BED (15 bupropion; 15 sertraline)	6 treatment	0%	Bupropion (150 mg/day) and (sertraline 200 mg/day) both reduced anxious-depressive symptoms and binge frequency. Bupropion more effective in reducing weight, with weight loss proportional to BMI, and improving sexual performances.	Randomized controlled trial
		Grilo et al., 2012b [55]	81 overweight with BED (27 fluoxetine, 26 CBT + fluoxetine; 28 CBT+ placebo)	4 treatment; 12 follow-up	28.4%	Remission rates at 12-month follow-up of 3.7% for fluoxetine-only, 26.9% for CBT + fluoxetine, and 35.7% for CBT + placebo. None of the treatments produced significant changes in BMI. CBT + fluoxetine and CBT + placebo did not differ from each other.	Double blind placebo controlled trial
		Guerdjikova et al., 2009 [76]	51 obese with BED (26 lamotrigine, 25 placebo)	4	44%	Lamotrigine (236+/−150 mg/day) and placebo performed similar on binge reduction, eating pathology, obsessive-compulsive symptoms, impulsivity, and global severity of illness. Lamotrigine was associated with a numerically greater amount of weight loss and significant reductions of glucose, insulin, and triglycerides.	Double blind placebo controlled trial
		Guerdjikova et al., 2012 [72]	40 BED with depression (20 duloxetine; 20 placebo)	3	32.5%	Duloxetine (mean 78.7 mg/day) superior to placebo in reducing binge frequency, weight and Clinical Global Impression-Severity of Illness ratings for binge eating and depressive disorders. Changes in BMI and measures of eating pathology, depression and anxiety did not differ.	Double blind placebo controlled trial
		Blom et al., 2014 [69]	10 articles (234 BED patients)	*-----*	*-----*	*Studying placebo response in BED, 38% of patients show partial response and 26% attain cessation. Lower baseline binge eating and longer study participation associated with higher response.*	*Pooled analysis of clinical trials*
		Leombruni et al., 2008 [70]	42 overweight with BED (22 sertraline; 20 fluoxetine)	6	35.7%	Sertraline (100–200 mg/day) and fluoxetine (40–80 mg/day) show similar efficacy on binge frequency, weight loss and psychopathology. Results were maintained by responders over 24 weeks.	Randomized double blind controlled trial
		Leombruni et al., 2009 [73]	45 BED fullcriteria or subthreshold	3	31%	Duloxetine (60–120 mg) reduces binges, eating impulsivity, depression, weight, BMI and clinical global impression.	Clinical trial
		Marazzitti et al., 2012 [75]	*------*	*-----*	*-----*	*Topiramate (25-1400 mg/day) and zonisamide (100-600 mg/day) seem to suppress appetite and increase eating control, leading to BMI reduction*	*Literature overview*
		McElroy et al., 2011a [77]	40 BED (20 acamprosate; 20 placebo)	2.5	38%	Acamprosate (999–2997 mg/day) improves binge frequency, obsessive-compulsiveness, food craving and quality of life.	Double blind placebo controlled trial
		Mc Elroy et al., 2011b [78]	12 BED	4	41.6%	Sodium oxybate (mean 7.1 g/day) reduces binge frequency, eating pathology, obsessive-compulsive symptoms, food cravings and weight.	Clinical trial
		McElroy et al., 2012 [68]	*22 articles*	*-----*	*-----*	*SSRIs are the most studied drugs effective on binge eating and psychiatric and weight symptoms. However most weight reductions would not be considered clinically significant. Duloxetine is effective on binge eating, weight loss and depressive symptoms. Reboxetine showed preliminary reductions in binge frequency and BMI. Topiramate is useful in BED with obesity, decreasing weight and obsessive-compulsive eating pathology as well as trait impulsivity. Orlistat in combination with CBT or dietary therapy, enhances weight loss and reduces binge eating. Zonisamide, naltrexone (high doses), stimulants, and glutamate-modulating agents show promises for BED treatment. Data are not sufficient to recommend pharmacotherapy as single first-line therapy, however drugs plays an important role in BED management.*	*Literature review*
		Reas & Grilo, 2008 [38]	*14 articles (1279 patients)*	*-----*	*-----*	*Pharmacotherapies have a clinical significance over placebo on binge remission and weight loss (statistically but not clinically significant). No data on long-term effects. Combining medications with psychotherapy failed to enhance outcomes. Promising findings, albeit modest, reported for orlistat/topiramate + CBT/BWL. Limited utility of SSRI. Need for additional large and longer studies.*	*Literature review*
Surgical interventions	4	Ashton et al., 2011 [84]	128 bariatric surgery candidates with binge eating	12 follow-up	-----	Positive responders to a brief CBT intervention targeting binge eating lost more weight both at 6 months and 12 postoperatively.	Longitudinal study
		Beck et al., 2012 [83]	45 bariatric surgery patients	24 follow-up	33% of eligible patients	Binge eating and ineffectiveness (self-administered tests) correlate with lower weight loss after surgery.	Retrospective study
		Faulconbridge et al., 2013^4^ [81]	85 obese with BED (36 bariatric surgery, 49 BWL)	12 surgical follow-up vs. 12 BWL treatment	41.2-17.6% to follow-up	Surgery vs BWL group treatment (20 weekly sessions + 10 fortnightly + 4 monthly sessions). Surgery participants lost significantly more weight. Improvements in mood and quality observed both in surgery and BWL intervention, with no differences at follow-up. Positive correlation between weight loss and change in depression scores. Weight loss at one time point predicted depression score at next time point, but depression score did not predict subsequent weight loss.	Longitudinal study
		Wadden et al., 2011^4^ [82]	144 surgically treated (59 no ED, 36 BED), 49 BWL intervention for obese with BED	12 surgical follow-up vs. 12 BWL treatment	41.2-17.6% to follow-up	At follow-up, surgically-treated participants without BED lost 24.2% of initial weight, compared with 22.1% for those with BED. Both groups achieved clinically significant improvements in several cardiovascular disease (CVD) risk factors. BED participants who received BWL lost 10.3% at 1 year. Mean binges number fell sharply in both BED groups at 1 year. Groups did not differ significantly in BED remission rates or in improvements in CVD risk factors.	Prospective observational study

*Studies published from January 2008 to May 2014 and concerning adult patients with BED. Review or meta-analysis are cited in italics in the table.

**Description of the trial upon which the study is based has been reported elsewhere [85].

1-2-3-4: Studies referring to the same patient sample.

Because of their limited usefulness in clinical practice, they were excluded 30 studies referring to neurobiological, neurocognitive and endocrine correlates of BED, 12 studies referring to laboratory tests and drugs not available in commerce, and 10 studies about diagnostic test validity. Twelve more excluded studies concerned secondary descriptive characteristics of BED, and 5 were about binge eating specificities among ethnic minorities. Five studies were not accessible to reading because written in languages different from English or Italian.

## Review

### Diagnostic issues and pathological features

According to the biopsychosocial model of mental diseases a comprehensive assessment and understanding of psychopathological mechanisms, maintenance factors, and dysfunctional areas of psychiatric illnesses are useful to support and address therapeutic interventions [23].

The concept of binge eating as “overeating without compensatory behaviors” was introduced by Stunkard in 1959, and structured in the Nineties by Fairburn and Spitzer as specific syndrome, mentioned in DSM-IV-TR as a nosologic entity needing future definition [24]. Due to an intense research activity providing solid evidence on the differences between BED and Bulimia Nervosa (BN), obesity and other ED-NOS [22,25], DSM-5 has recently recognized BED as an autonomous disease, maintaining diagnostic criteria which are consistent with the previous edition, but reducing both frequency of the binging episodes and duration of eating behaviors required to make the diagnosis [1].

In addition to diagnostic criteria included in DSM that have proved clinical relevance and diagnostic efficacy in several studies [22,26], also other psychopathologic basic features have been outlined as relevant for treatment planning and outcome. Overvaluation of shape and weight is one of these conditions. It is present in 60% of BED patients, a level which is significantly higher than in non-BED obese patients, even though it is lower than in AN and BN, where it is nearly universal [27]. Even if this feature is present only in a subgroup of BED patients, and so it is not included among the official diagnostic criteria, it is related to higher psychosocial impairment and worse quality of life [27]. BED patients frequently refer to a constant polarization of thoughts on weight control, diet and binge-avoidance, showing worst eating control, higher fear of weight gain, and higher body-shape dissatisfaction than non-BED obese individuals, even though lower than in BN subjects [16,21,25]. Overvaluation of shape and weight can be thus considered either a diagnostic specifier or a dimensional severity rate, with a primary relevance for diagnosis, treatment choice, global impairment and outcome, and it can also be considered as a specific target for psychological therapies [10,27,28].

Also food intake patterns are clinically important in BED, as showed by the pathogenic model which has been proposed for this disorder - inspired to the one proposed by Fairburn and Cooper for BN. According to this model, low self-esteem, body image-related, generates anxiety and negative emotions resulting in excessive diet restrictions, in turn triggering bingeing [29]. Obese individuals with BED may lack effective strategies for managing negative emotions, and it has been hypothesized that eating is undertaken as a strategy to regulate or change negative emotions [30]. Recent studies, however, describe binge eating in BED as the possible result of an immediate breakdown of emotion and impulse regulation caused by sudden increases of negative affect and tension, and/or rapid decrease of positive affect. Instead, it is unlikely that such behavior is consequent to gradual accumulation of negative mood and subsequent immediate relief as observed in BN [31]. Therapeutic implications of these findings are represented by the development of response-prevention strategies, such as the acceptance of stressful events or the disclosure of mental states. In contrast the suppression of unwanted behavior during of high mental load may increase the probability of bingering behavior [31].

In BED patients, daily distribution of food intake shows peculiarities, unrelated to mood fluctuations, which should be taken into account to process better dietary and behavioral strategies to manage symptoms and achieve therapeutic goals [32]. In fact, in BED patients they have been evidenced significantly more atypical eating behaviors, like dietary restraint or morning under-eating, or recurrent overeating patterns like eating double meals, or nocturnal eating and snack consumption [18,32]. On the other hand, more frequent breakfast consumption correlate with lower BMI and more frequent meal consumption with reduced binge eating [32].

The majority of binge episodes typically occur in less than 2 hours, with a clinical and psychopathological importance which is related to objective food intake [33]. Loss of control is a core feature associated with higher depression, higher body dissatisfaction and poorer related quality of life [33]. The peculiar food choice that patients make during binge episodes - e.g. fats, carbohydrates, sweets and snacks, − has been related to the hypothesis of a “hedonic deprivation”, were eating impulsiveness can be triggered by restrictions on palatable foods during everyday life, even if at more recent findings the presence of this clinical feature seems controversial [33].

Linked to neurobiological mechanisms similar to those of substance abuse, also “food dependence” is an important correlate of this finding, with dopamine, serotonin, and endogenous opioids systems playing a role in this regard [16]. Patients with BED show an increased reward sensitivity towards food and increased rash-spontaneous behavior, thus shaping a phenotype of obesity with increased impulsiveness which should be identified and focused by specific psychological or pharmacological treatment [16]. Recent studies underline indeed the importance of genes on eating habits, identifying a heritable component of medium-high importance, referred to the whole category of ED and with a gender-independent transmission [34].

Regarding to psychopathological peculiarities and clinical implications, BED should be also differentiated from other obesity-related eating patterns characterized by eating impulsiveness, not yet recognized as autonomous syndromes but in a number of cases present in those affected by BED [32]. Some these conditions are snacking (introduction of small amounts of food throughout the day), emotional overeating (eating in response to intense emotional states), and selective cravings (intense desire and consuming of specific foods, e.g. sweet eating), that should be considered as subtypes of binge-eating; however, their clinical implications have to be defined yet [25,30,31].

### Treatment

#### Multidisciplinary sequential approach

The treatment of BED, as for EDs in general, is influenced by his etiological background, constituted of a complex interaction of heritable, psychological and environmental factors that should be taken into account in treatment planning [23,35]. Treatment choices should then be multidisciplinary, suited to cope with symptoms and comorbidities and also with the high drop-out rates and the low maintenance of achieved results typical of this disease [36,37]. Hallmarks of BED are in fact low self-esteem experiences, weak therapeutic alliance, low mastery and clarification experiences, in turn predicting treatment dissatisfaction with and early discontinuation of care [37]. To deal adequately with these features and with the different core psychopathologic traits, the therapeutic setting should be flexible and considering exhaustively psychological and physical impairments [23,35]. Moreover, the decision to seek treatment should be preceded by enhancing patients’ motivation on their condition [23]. The therapeutic project should be tailored on the patient in accordance with protocol recommendations and it should be frequently revised, on the basis of the clinical needs [23]. Literature evidences concerning the combination of different treatment strategies at the same time are weak [35,36,38,39], instead it seems more promising to provide stepped-care treatments with rising levels of intervention intensity tailored on disease severity [40-43].

Primary goal of BED treatment is to achieve abstinence from binge eating, and afterward a sustainable weight loss [44,45]. Nevertheless treatment should also target the increase and maintenance of motivation, the education to healthier eating and life styles, the modification of dysfunctional thoughts and habits, the increase of insight and abilities to deal with conflicts and negative emotions, the treatment of physical and psychiatric comorbidities and relapse prevention [23,36,43,46]. The therapeutic program should promote a stable reduction of caloric intake and a permanent maintenance of healthier eating and lifestyle habits, including instructing patients to self-monitor their symptoms [36,43]. More encouraging results can be obtained indeed with patients trained to manage autonomously symptoms, mood fluctuations, anxiety and stress levels [43,46]. This training seems to be helpful in making the patients able to recognize their needs, modify their thinking style, and facilitate not only their cognitive but also their experiential and intuitive learning [36,43,46]. To enhance efficacy on weight loss, which is not a primary outcome of BED treatment but a factor associated with consistent impairment and comorbidity, some evidences support the association of pharmacological and psychotherapeutic treatments to behavioral interventions, based on weight and diet monitoring, and borrowed by general obesity management [35,36,38,40,47].

It can be useful to set an adequate treatment plan as soon as possible, because early responses to treatment are important from a prognosis standpoint [43,45,48]. That can be favored by treatment specificity and stepped-care treatments, providing periodic re-evaluations addressing non-responders to more specialized interventions [41-43].

Binge abstinence should be pursued at the very beginning of treatment, also because the achievement of this goal per se in some cases leads to significant weight loss [44]. Nevertheless in most cases treatment should not target weight loss at first, but weight stabilization [44,49]. Some studies in fact show frequent and substantial weight gain one year prior treatment seeking, providing an important element to interpret the modest weight losses typically reported in literature [49,50]. Also weight regain is a critical target for BED patients, who report marked weight fluctuations and spend much time trying to lose weight [50]. The poor weight loss showed in literature studies may than be re-interpreted at the light of a successful weight stabilization, and an effective prevention of further weight regain [49,50].

#### Behavioral interventions

Behavioral treatments (BWL), focused on diet and lifestyle modification, and borrowed by obesity treatment, have frequently been proposed as useful basic interventions for BED treatment that showed some appreciable results which are comparable to more complex therapies, in patients with low associated psychopathology [44,51].

Diet therapy is essential to promote weight loss, and for this reason it is object of great attention among BED patients who are characterized by high body dissatisfaction [21] and higher overvaluation of body weight and shape, which generally represent negative outcome predictors [10]. Moreover it should be reminded that, even if body weight is not directly linked with disease severity, it is strictly correlated with several medical comorbidities [4]. Furthermore, body weight heavily influences perceived wellness, mood and self-esteem in those affected by BED and, consequently, binge vulnerability itself [27,18]. On the other hand binge episodes are recurrently triggered by excessive dietary restraint which can be enacted by BED subjects to reduce body weight faster [18,29]. Therefore, prescribed caloric restrictions in BED should be moderate, and a normal or hypocaloric diet seems to be the best strategy to prevent bingeing [40,46]. Weight loss anyway should be pursued only after binge reduction or stabilization and the attempt to lose considerable weight in a short time should be discouraged, because of the increased risk of weight regain [49,51,52]. Poor compliance to diet should not be blamed but it should be addressed instead when planning the nutritional scheme, because only its management could make it possible to reach a stable control over eating behaviors [40]. It is recommended not to totally exclude any food choices; it could be more useful instead to encourage patients to moderate their food intakes according to individual needs [40].

Physical activity, coupled to a balanced diet, is another basic factor for weight loss, both for its direct action on fat consumption and its inhibitory action on excessive food intake [47]. It also helps maintaining goals reached with diet, and it improves mood and general health, lowering clinical comorbidities of BED [47]. However, due to frequently compromised health conditions of BED patients, physical activity should be established gradually and under close medical supervision [47]. In conclusion, it must be anyhow underlined that, even if BWL therapies have showed in some studies a relevant usefulness, especially on weight loss [35,36,44,52], their efficacy in BED remission is lower than more specific intervention like psychotherapies, and that their dropout rates are higher [43,46,52].

#### Psychoeducational interventions

Psychoeducational treatments for BED are purposed to inform patients about different aspects and correlates of their disease, in order to promote a deeper comprehension of their condition and thus to increase the possibilities to manage it [53,54]. These interventions give information about core topics like the factors that predispose, trigger and maintain eating impulsiveness, negative effects of overweight and unbalanced or restrictive diets, and most effective methods of weight regulation [53]. Treatments are focused on plain symptoms explanation, without adopting strategies to influence unconscious or cognitive maintaining factors at a deep level, unlike to what occurs in psychotherapies [53,54].

Advantages of these treatments are that they can be administered by low-specialized personnel, they can be easily manualized, and they can be structured in weekly group meetings of limited duration [53]. Patients are taught to self-monitor food intake and are informed about factors that trigger binges and about correct lifestyles, sustainable in time [53,54]. They are also explained the pathogenic mechanisms of the disease and about strategies suitable to establish a stable change. Interventions are focused on lifestyle modification to promote a general improvement of health and quality of life, rather than on weight loss itself [53,54].

These interventions have showed a preliminary efficacy on binge reduction and eating impulsivity [53,54], even if effects on body dissatisfaction, anxiety, depressive symptoms and weight loss are still controversial [54]. Among patients who undergo this kind of intervention, higher eating impulsivity correlates with higher drop-out rates, and this suggests that more specific and intensive intervention should be suitable for patient with heavier psychopathology [53].

Considering these preliminary findings, psychoeducational interventions could constitute useful baseline therapies that constitute a useful starting point for more complex treatments, and that can show efficacy comparable to more complex interventions on less severe cases. Studies with randomized control groups, are still needed to support this recommendation [53].

#### Psychotherapies

According to literature data, also with regards to systematic reviews and meta-analysis, psychotherapies are the most validated and effective treatments for BED [36,38,43].

Cognitive-Behavioral Therapies (CBT) are the most evaluated and developed psychological intervention for treating BED [36,38]. The focus is the etiological basis of bingeing and its relation with a self-esteem excessively dependent form body shape [10,38]. The most widely used CBT models are adaptations of those developed for BN, paying close attention to weight loss and taking into account the lower restriction levels and the lower cognitive distortions of BED compared to BN [38,43].

CBT is a practical and adaptable intervention, with setting and duration that can be tailored on clinical needs and the possibility of being carried out independently or in combination with other pharmacological or BWL approaches [36,38-40,47]. Anyway evidences supporting combination treatment are still limited and controversial, while CBT have yet showed high efficacy when administered alone, with BED remission rates around 50-60% [9,52,55]. Even if in BED CBT failed to show higher immediate binge reduction when compared to BWL interventions at short-time observations, it seems to overcome these therapies especially over long periods of time, improving quality of life, and reducing drop-out rates [44,52]. Long term effects of CBT approaches include, in fact, a gradual normalization of eating patterns and reduced relapse occurrence, these effects are coupled with the improvement of disinhibition, hunger, negative feelings and perceived global health [51,56,57]. Unfortunately, evidences about effects on weight reduction are still limited and of unclear clinical significance [9,36,52]. Also data about advantages of coupling BWL interventions targeting weight loss still needs further deepening [40,52]. Individual or group setting have showed a similar efficacy, maintained at 3 years follow-up [43,57]. Instead long-term versus short-term interventions have showed comparable results on patients with favorable outcome predictors, but it was evidenced an higher efficacy of long-term treatment in patients with higher psychopathology [58].

Among outcome predictors, literature data have showed a consistent importance of a rapid response to treatment, of lower overeating frequency, and of lower general psychopathology levels, but also of higher social embedding and higher openness [44,59].

CBT-based treatments have showed their usefulness also when administered in self-help setting, thus offering another valid first-line treatment option which is more efficient than treatment as usual [60], superior to BWL-based self-help models [43], and without contraindications for patients with comorbid conditions [61]. From a cost-effectiveness standpoint self-help interventions constitute a valid first-line option while more specific therapies like IPT or CBT could be used in a more specific way for patients with higher eating psychopathology and lower self-esteem [43]. Self-help interventions can be guided by either therapists trained in EDs (who can run all sessions or only some), or not ED-specialized therapists or managed by patients only [62,63]. The presence of a trained therapist who leads the group sessions gives a better short-term outcome and less group contrast, but significant differences have not been showed so far as regards outcomes between guided and self-help groups at follow-up [62,63]. Some preliminary studies have also evaluated the efficacy of Internet guided self-help programs, but their efficacy is still debated [62].

Dialectic-Behavioral Therapy (DBT) is another psychotherapeutic approach that has proved its efficacy in BED; it is more focused on emotion regulation and stress tolerance than CBT [43]. This therapy showed to be effective in binge reduction and in lowering concerns about food and body shape similarly to CBT, but it has not provided clear results on weight loss, depression, or anxiety [64,65]. It seems to be effective also when negative outcome predictors occur as earlier age at binges onset and higher personality disturbances [7,48]. Some preliminary findings show DBT to be effective also when administered as a self-help intervention, a suitable low-intensity treatment option for BED [66].

Another evidence-based psychological treatment is Interpersonal Psychotherapy (IPT). This technique focuses on personal relations and role transitions that could have a predisposing and maintaining role in EDs, in order to achieve better social interactions and to cope with interpersonal conflicts [41-43]. Even if it doesn’t focus directly eating symptoms, it can be useful because it targets a specific area of impairment in BED patients [42]. This disease in fact often emerges in adolescence [4], in a context of interpersonal and maturation difficulties, and its maintenance over the years can be related to dysfunctional relational styles that triggers depression, anxiety, and anger feelings in turn underlying eating impulsiveness [11]. It has been hypothesized that IPT, also administered in a psychodynamic framework, with additional focus on cyclical dysfunctional relational patterns, could be particularly effective on individuals for whom negative moods act as major triggers for bingeing or with cold/distant attachment style [41]. It appears in any case effective on depression and psychosocial discomfort [41,43]. It has been observed that CBT and IPT have comparable efficacy in binge reduction, both at the end of therapy and at one-year follow-up, with a significant reduction of psychiatric comorbidities and in some cases a significant decrease in body weight. Therapeutic results are stable at a 4-year follow-up [67,42,43].

All in all, psychotherapeutic approaches to BED, mostly based on CBT models, are useful first-line treatments, even more than pharmacological approaches [36,43].

#### Pharmacological treatment

Pharmacological therapy in BED is specifically focused on the reduction of eating impulsiveness, binges and negative feelings, constituting a co-cause and a complication of eating symptoms. It has been noticed a statistically significant drug action on short-term binge remission and also on weight loss, although this does not hold significant at longer follow-up [36,38]. Unfortunately, few data exist on long-term efficacy of pharmacotherapy as stand-alone treatment for BED, and in some studies combined treatment of drugs and psychotherapy interventions failed to enhance outcome significantly [55,35]. Most treatment options currently used in BED have at first demonstrated their efficacy on binge reduction in BN, but non-psychoactive drugs, borrowed from obesity treatment, are also in use in selected cases or in combination with other treatments like psychotherapies [68].

As an additional element supporting the specificity of BED diagnosis, it is noteworthy that psychopharmacological therapy showed higher efficacy in BED than in non-BED obese population and that placebo response was consistent with the one observed in other major psychiatric diseases [69].

Antidepressants are the most widely studied and applied medications in BED treatment, showing efficacy on eating impulsiveness and eating and general psychopathology, but also on anxiety and depressive symptoms, with supposed secondary positive effects on eating impulsiveness due to lowering of negative affects that trigger binges [36,38].

In any case it is recommended to use compounds showing side effects on weight and hunger, and it is better to choose pro-anorectic antidepressants molecules to improve treatment compliance. It is for this reason that tricyclic antidepressants are not commonly used in EDs, while selective serotonin reuptake inhibitors (SSRIs) are preferred given their anti-impulsive action [68]. On the other hand, when eating impulsiveness is not related to a specific psychopathology, it is possible to evaluate in selected cases the use of medications that act directly on hunger regulation and food absorption, without influencing mood [36].

SSRI at high doses have showed efficacy on binge reduction and associated psychopathology, with some limited evidences of effectiveness on weight loss, which are of questionable clinical significance [38,68]. Even if differences in long-term action among SSRI still have to be clearly established, fluoxetine is the most widely studied and prescribed because of its registration for BN [70]. It proved significant efficacy above placebo on binge reduction, weight loss and mood improvement [69,70], but uncertain clinical relevance and consistently lower effects than CBT, also in combination treatments [55,70]. Sertraline and fluvoxamine have showed similar results to fluoxetine, while citalopram and paroxetine are scarcely implied because of their side-effects on hunger and weight gain [68,70,71].

Duloxetine in some initial studies was reported to reduce hunger and binges and promote weight loss [72,73], even the current body of evidence is still lacking [68].

Reboxetine showed significant reductions in binge frequency and BMI in a small preliminary open-label trial of 12-week [67].

Bupropion showed effectiveness on binge eating, anxiety, and depressive symptoms in a single study, with better efficacy than sertraline on sexual side-effects and weight loss [71].

Among anti-epileptic drugs, topiramate reduced hunger, promotes weight loss, and can obtain a significant reduction in daily and weekly binge episodes and impulsivity. Nevertheless, it has not showed efficacy on psychopathological distress or depressive symptoms, while its results on weight loss are still debated [68,74,75]. Lamotrigine showed preliminary efficacy on weight loss and metabolic dysfunction but without results on general and eating psychopathology, and global severity of illness [76]. Also zonisamide seems to be able to suppress appetite and increase eating control, leading to BMI reduction [75,68].

Psychostimulants like atomoxetine, naltrexone and glutamate-modulating agents seems to be promising treatments, acting on binge reduction and weight loss when compared to placebo [68].

Acamprosate was associated at endpoint analysis with improvements on binge frequency, food craving and quality of life compared to placebo [77]. Sodium oxibate, in a small sample without control group, show reductions of binge episodes, related psychopathology, and also weight loss [78].

In conclusion, current literature data evidence still unclear efficacy of pharmacological treatments for BED. Nevertheless they are widely applied in clinical practice and play an important role in BED management [68]. Even though these treatments produced a significant reduction of binges and eating impulsiveness, and in some cases also weight losses, clinical relevance of these modifications is still doubtful and limited by small sample sizes, high dropout rates, and short follow-up times [38,68]. Also the usefulness of pharmacotherapy as association treatment, with the purpose to enhance effects of BWL or psychotherapies, is still highly unclear, with scarce data and discordant findings at support [35,36,38,68].

#### Surgical interventions

Bariatric surgery is a recommended treatment for severe obesity (BMI ≥40 or BMI ≥35 with comorbid conditions) according to current guidelines [79], while it is not mentioned among evidence-based BED treatments [80]. However obese individuals with BED frequently experience weight-related impairments in mood and quality of life, which improve with weight loss [19]. This improvement of wellness and quality of life acts as a protective factor against binge vulnerability, making bariatric surgery a useful option in severe obesity linked to BED [81]. The high disability linked to BED itself, coupled with the difficulty to achieve a stable weight loss with traditional behavioural interventions, causes an elevate BED prevalence in surgery candidates, ranging from 5% to 50% [82]. On the other hand, an ED diagnosis was once considered a major contraindication to bariatric surgery. This limitation has been reduced over time and bariatric surgery interventions can now be suitable for selected BED patient, even if the extent of weight loss depends on the presence of binge episodes after the intervention [83].

Faulconbridge and coworkers [81] explored the effects of bariatric surgery in BED patients with severe obesity, trying to enlighten if observed improvements are due to weight loss itself or to additional aspects of treatment, such as therapeutic support or acquisition of cognitive-behavioral skills. They found significant improvements in mood and quality of life at follow-up both in patients who undergo surgery and in controls enrolled in a BWL intervention, but they evidenced no differences between the groups at month 12. Nevertheless, bariatric surgery showed a significantly higher efficacy on weight reduction [81].

Wadden and coworkers [82] found no relevant differences in post-surgical weight loss between patients with and without BED, higher than weight loss obtained with lifestyle modifications at 1 year follow-up. The mean number of binge eating days fell sharply both with surgery and with BWL interventions [82].

Nevertheless, due to the required changes in patients’ lifestyle and the high economic costs of bariatric surgery, candidates should be carefully screened. These treatments should then be proposed after a careful evaluation of psychological conditions, and a psychiatric and psychological assessment before and after surgical intervention is recommended. In fact, bariatric surgery or lifestyle modification treatments led to comparable results on mood and quality of life at the 1-year follow-up [84]. To achieve good outcome it is fundamental to provide the patient with a framework of nutritional-metabolic and psychological rehabilitation, focused on empowering and stimulating an active and informed collaboration [84]. In the first months after surgery, patients with BED who present a high gastric capacity before intervention can show an initial weight loss even higher than usual and complete binges remission [82]. However binge behaviors tend to resume partially after 12 months follow-up [82]. Binge recurrence may occur when patients learn to manage their new condition, and to circumvent eating limitations caused by the intervention – e.g. replacing classical binge episodes with great caloric intakes in liquid or semi-liquid forms [82,84].

It is noticeable from these data that a stable binge control, obtained with pharmacological o psychological intervention, should be a primary outcome to be pursued in BED patients that undergo bariatric surgery [83]. It predicts better prognosis which is frequently not significantly different from that of non–BED obese individuals [81,82,84].

## Conclusions

Despite its recent inclusion in DSM-5 as an autonomous disease, further research is needed on BED diagnosis and treatment strategies, through larger samples and longer follow-up times [36]. Although present diagnostic criteria has showed their empirical consistency other core psychopathologic features like body and weight overvaluation may be of clinical and prognostic relevance [27]. Binge stability and spontaneous remission rates should be better explored [9], deepening the identification of prognostic factors able to address treatment choices and to favor more specific and cost-effective interventions [36,37]. Also the importance of comorbidities and weight maintenance in treatment adherence and remission should be extensively investigated because of its influence on prognosis and treatment [18].

Multidisciplinary treatment choices seems to emerge as the best treatment strategy for long-term management of this disease, with primary goals on binge abstinence and at a second time a sustainable weight loss [44,45]. Nevertheless treatment should also target secondary outcomes as the increase and maintenance of motivation, the reduction of drop-out rates and the management of relapses [43,46]. Further studies are however required to enlighten most promising treatment combinations, considering evidences of limited usefulness of combining different treatments at the same time [35,36,38,39]. Stronger literature support stepped-care treatments, with a rising intensity of intervention tailored on disease severity [40-43].

Psychotherapeutic approaches to BED, mostly based on CBT models, are recommendable as first-line treatments with the wider evidences of efficacy also at long-term follow-up, but still unclear results on weight loss [36]. Otherwise simpler and cheaper interventions like BWL treatments, psychoeducational interventions and self-help treatments have showed significant efficacy in patients with lower disease severity and less comorbidity [43,44,53].

Drug therapy, especially SSRIs can be useful in lowering eating impulsivity and improving psychiatric comorbidities [36]. Unfortunately, long-term efficacy of pharmacotherapy as a stand-alone treatment for BED needs further research, as well as long-term effects of integrated treatment combining drugs and psychotherapy [36,38].

Even if bariatric surgery is not a recommended treatment for BED according to current guidelines [79], its clinical relevance in BED coupled with severe obesity deserves more careful clarifications [80].

In summary, even if literature data about BED diagnosis and treatment are gradually deepening and improving in detail and significance, further studies, especially literature review and meta-analysis, are still required with major regard to long term outcomes, stepped care treatment, improving of weight loss (also with surgery treatment) and lowering dropout rates.

## Abbreviations

BED: Binge Eating Disorder
EDs: Eating disorders
ED-NOS: Eating Disorder not Otherwise Specified
BMI: Body mass index
AN: Anorexia nervosa
BN: Bulimia nervosa
DSM: Diagnostic and Statistical Manual of mental disorders
CBT: Cognitive behavioral therapy
BWL: Behavioral Weight Loss
DBT: Dialectic-Behavioral Therapy
IPT: Interpersonal Psychotherapy
SSRI: Selective Serotonin Reuptake Inhibitors
NARI: Noradrenalin and Adrenaline Reuptake Inhibitors
SNRI: Serotonin and Noradrenalin Reuptake Inhibitors
